# Translationally controlled tumor protein interacts with connexin 43 and facilitates intercellular coupling between cardiomyocytes

**DOI:** 10.3389/fcell.2025.1549063

**Published:** 2025-03-20

**Authors:** Yaopeng Hu, Wenqian Cai, Yuko Hidaka, Keizo Hiraishi, Jiehui Cang, Masanari Umemura, Utako Yokoyama, Björn C. Knollmann, Yoshihiro Ishikawa, Takayuki Fujita

**Affiliations:** ^1^ Department of Physiology, Fukuoka University School of Medicine, Fukuoka, Japan; ^2^ Heart Center and Institute of Pediatrics, Guangzhou Women and Children’s Medical Center, Guangzhou Medical University, Guangzhou, China; ^3^ Department of Physiology, Tokyo Medical University, Tokyo, Japan; ^4^ Cardiovascular Research Institute, Yokohama City University Graduate School of Medicine, Yokohama, Japan; ^5^ Center for Arrhythmia Research and Therapeutics, Vanderbilt University School of Medicine, Nashville, TN, United States

**Keywords:** translationally controlled tumor protein, Cx43, heart, gap junctional intercellular communication, cardiac arrhythmias

## Abstract

**Introduction:**

Connexins are gap junction proteins that play pivotal roles in intercellular communication. Connexin 43 (Cx43) is one of the most ubiquitously expressed connexin isoforms in human. Cx43 has been demonstrated to be involved in the pathological process of various diseases, including arrhythmias. Recently, translationally controlled tumor protein (TCTP), a highly conserved anti-apoptotic protein, has been shown to play an important role in protecting against the development of heart failure. However, its role in arrhythmogenesis remains unclear. In this study, we aimed to examine the interaction between TCTP and Cx43 and investigate the roles of TCTP in the formation of Cx43 gap junction channels and gap junctional intercellular communication (GJIC) in cardiomyocytes.

**Methods and results:**

We found that TCTP was predominantly expressed in the intercalated discs of mouse heart tissue. Cx43 in adult mouse hearts was coimmunoprecipitated using a TCTP-specific antibody. Additionally, co-localization of TCTP and Cx43 was demonstrated using a proximity ligation assay in iPS cell-derived human cardiomyocytes. TCTP silencing reduced the formation of Cx43 gap junction channels at the intercellular contacts between cardiomyocytes. Moreover, TCTP silencing significantly attenuated GJIC among cardiomyocytes. Interestingly, the development of ventricular arrhythmia was attenuated in cardiomyocyte-specific TCTP-overexpressing mice.

**Conclusion:**

These findings indicate that TCTP regulates GJIC. Thus, TCTP may be a therapeutic target for preventing Cx43-related pathogenesis.

## Introduction

Translationally controlled tumor protein (TCTP) is a multifunctional molecule ubiquitously expressed in mammalian tissues. TCTP plays critical roles in various important biological processes, including cell growth, development, carcinogenesis, cell death, and immunological responses (Telerman and Amson, 2009; Bommer and Telerman, 2020). At present, TCTP is attracting interest as a causative factor of clinically significant diseases and a therapeutic target. Indeed, TCTP has been reported to be involved in the development of malignant tumors, heart failure, systemic hypertension, pulmonary arterial hypertension, atherosclerosis, and bronchial asthma (Bommer and Telerman, 2020; Chen et al., 2011).

We recently reported the crucial role of TCTP in the heart (Cai et al., 2019). TCTP plays an important role in the viability of cardiomyocytes. TCTP protects against cardiomyocyte death by inhibiting apoptosis and autophagy. Consistently, TCTP overexpression protected against doxorubicin-induced heart failure in mice. Although previous reports have demonstrated that TCTP is expressed in the plasma membrane, nucleus, and mitochondria and plays important roles in various cellular processes (Acunzo et al., 2014; Amzallag et al., 2004; Cai et al., 2019), the functions of TCTP in gap junctions remain to be investigated.

The gap junction channel consists of two docked hemichannels, each located on the plasma membrane of different adjacent cells. The channels serve as pathways between cells and facilitate direct intercellular communication through which less than 1 kDa molecules, including ions, ATP, cAMP, inositol 1,4,5-trisphosphate, and other nutrients and metabolites, can be transferred (Saez et al., 2003; Veenstra et al., 1995). Connexins are component proteins of the connexons and consist of 21 isoforms in humans. Connexins are involved in the pathological processes of various diseases, including arrhythmias, heart failure, hypertension, ischemia/reperfusion injury, depression, and neurodegenerative diseases (Zhang et al., 2023). Connexin 43 (Cx43) is one of the most ubiquitously expressed isoforms across human tissues and the most prevalent connexin isoform in the heart (van Kempen et al., 1991). Cx43 is involved in various important functions in cardiomyocytes and other cell types including astrocytes, epithelial cells, and vascular smooth muscle cells (Rodreguez-Sinovas et al., 2021; Leybaert et al., 2023; Zhang et al., 2023).

In this study, we examined the relationship between TCTP and Cx43 expression in the heart. Gap junctional intercellular communication (GJIC) at intercalated discs plays a pivotal role in the conduction of action potentials in the heart and is essential for the maintenance of harmonized cardiac contraction. The attenuation of coupling is thought to cause slow conduction, which is crucial for the development of reentrant arrhythmias. Elevated vulnerability to arrhythmia was observed in mice with reduced Cx43 expression (Lerner et al., 2000). Additionally, it has been reported that Cx43 is involved in the maintenance of Nav1.5 expression, thereby regulating the conduction velocity of the action potential (Jansen et al., 2012a). Additionally, the role of Cx43 interacting proteins including zonula occludens-1 (ZO-1) (Dai et al., 2020; El Refaey et al., 2022; Hunter et al., 2005) and vinculin (Zemljic-Harpf et al., 2007; Cheng et al., 2017; Zemljic-Harpf et al., 2014) in the regulation of connexin expression at intercellular contacts and the development of ventricular ectopy have been reported. Therefore, the regulation of Cx43 function at intercellular contacts is considered a promising therapeutic target for arrhythmias. Hence, elucidation of the regulatory mechanism of Cx43 gap junction channel formation and intercellular communication through these channels is important.

In this study, the experiments showed that TCTP was predominantly expressed in intercalated discs in the mouse heart. Cx43 in adult mouse hearts was coimmunoprecipitated using a TCTP-specific antibody. Using a proximity ligation assay, we demonstrated the close proximity colocalization of TCTP and Cx43 in cardiomyocytes. Moreover, TCTP silencing reduced the expression of Cx43 at intercellular contacts and significantly attenuated GJIC between cardiomyocytes. These findings suggest that TCTP regulates GJIC. Additionally, cardiomyocyte-specific overexpression of TCTP attenuated the development of catecholamine-induced ventricular arrhythmia in mice.

## Materials and methods

### Reagents and antibodies

The TCTP antibody (PM107) was purchased from Medical and Biological Laboratories Co., Ltd. Anti-Cx43 antibody (MAB3068) was purchased from Millipore. Anti-GAPDH antibody (sc-32233) was purchased from Santa Cruz Biotechnology. Anti-Cx43 antibody (C8093) and isoproterenol (ISO) were purchased from Sigma-Aldrich. Anti-β-actin antibody (4967) was purchased from Cell Signaling Technology. Anti-N-cadherin antibody (MA5-15633), calcein-AM (C1430), and lucifer yellow (L453) were purchased from Thermo Fisher Scientific.

### Cell culture

#### Neonatal rat ventricular myocytes (NRVMs)

Primary NRVM cultures were prepared as previously described (Cai et al., 2019; Okumura et al., 2014). The hearts from 1–3-day-old Wistar rats (Japan SLC Inc.) were minced and dissociated with 0.014% type II collagenase (Worthington), DNaseI 0.0015% (Roche) and 0.04% pancreatin (GIBCO). The dispersed cells were plated on 10 cm dishes for 70 min to remove fibroblasts. Non-attached cells were collected and seeded onto gelatin-coated dishes. Cells were cultured in Dulbecco’s modified Eagle’s medium (DMEM/F12) containing 10% fetal bovine serum and a 1% solution of penicillin-streptomycin at 37°C in 5% CO_2_. The next day, the medium was replaced with a serum-free medium.

#### Human-induced pluripotent stem cell-derived cardiomyocytes (iPSC-CMs)

iPSC-CMs (ax2508) were sourced from Axol Bioscience (Little Chesterford, Cambridge, United Kingdom). These iPSC-CMs were originally generated from pulmonary fibroblasts of a male donor using an episomal vector. Cryopreserved cardiomyocytes were rapidly thawed and resuspended in a maintenance medium supplemented with Axol’s proprietary additives (ax2530) and 10% fetal bovine serum. The cells were subsequently seeded into dishes pre-coated with fibronectin coating solution (ax0050). After 24 h, the plating medium was replaced with a serum-free maintenance medium for the first time and subsequently refreshed every other day.

### Transfection of small interfering RNA (siRNA)

Both double-stranded TCTP siRNA and control siRNA were purchased from Dharmacon. The TCTP siRNA sequence was UGGUUGCUCUACUGGACUA. NRVMs were transfected with siRNA using Lipofectamine RNAiMAX Transfection Reagent according to the manufacturer’s instructions (Invitrogen).

### Immunocytochemistry

#### For cultured cells

Primary NRVMs were seeded on cell- and tissue-adhesive (Corning Cell-Tak™)-coated coverslips. Cells were washed twice with ice-cold phosphate-buffered saline (PBS) and then fixed in 4% paraformaldehyde at room temperature for 15 min, followed by permeabilization with 0.2% TritonX-100 for 5 min. After washing with PBS three times, cells were blocked with 5% bovine serum albumin in PBS for 1 h. Cells were incubated with primary antibodies at 4°C overnight, followed by 1 h incubation with secondary antibodies (goat anti-mouse AlexaFluor 488 or goat anti-rabbit AlexaFluor 594, Molecular Probes for NRVMs; goat anti-mouse AlexaFluor 568 or goat anti-rabbit AlexaFluor 488, Invitrogen for iPSC-CMs), and 5 min incubation with 4′,6-diamidino-2-phenylindole (DAPI). Images were captured using a Zeiss LSM 710 Confocal Microscope (Oberkochen, Germany).

#### For tissue section

Mouse hearts were fixed with formalin, embedded in paraffin, and sectioned at 3.5 μm. Sections were deparaffinized and incubated in 0.3% H_2_O_2_ in methanol for 30 min to inactivate endogenous peroxidases. Then incubated in citrate buffer (pH 6.0) at 100°C for 10 min. Following blocking in 5% skim milk for 30 min at room temperature, sections were incubated with primary antibodies at 4°C overnight. After 1 h of incubation with the secondary antibody, tissues were processed by the ABC method using a commercially available kit (Vector Laboratories, Burlingame, CA) or a 3,3-N-diaminobenzidine tetrahydrochloride kit according to the manufacturer’s instructions.

### Mice

We generated TCTP transgenic mice (TCTP TG) with cardiac-specific overexpression of TCTP using α-myosin heavy chain promoter on a C57BL/6 background (Cai et al., 2019). The calsequestrin 2 gene knockout (Casq2 KO) mice (Knollmann et al., 2006) were kindly provided by Dr. B. C. Knollmann (Vanderbilt University School of Medicine, Nashville, TN, United States). TCTP TG-Casq2 KO mice were generated by intercrossing TCTP TG and Casq2 KO mice; age-matched Casq2 KO mice from the same colony were used as controls.

### Induction of ventricular arrhythmias

Catecholaminergic ventricular arrhythmias were induced by catecholamine challenge in Casq2 KO or TCTP TG-Casq2 KO mice, as previously described (Knollmann et al., 2006; Suita et al., 2018; Prajapati et al., 2019). The mice were anesthetized with isoflurane (1.5%) inhalation, and ISO (3 mg/kg) was injected intraperitoneally. The number of premature ventricular contractions (PVCs) was counted using a lead II body surface electrocardiogram (ECG) for 20 min after ISO administration.

### Co-immunoprecipitation (Co-IP)

Proteins were extracted from the mouse heart left ventricle tissues homogenized in an IP Lysis Buffer (50 mM Tris-HCl [pH 7.5], 150 mM NaCl, 5 mM ethylenediaminetetraacetic acid, 1% NP-40) containing a protease inhibitor cocktail with complete ethylenediaminetetraacetic acid (EDTA)-free (Roche). Tissue lysates were centrifuged at 15,000 rpm for 15 min at 4°C. The tissue lysis was incubated with TCTP antibody (PM107, MBL) overnight at 4°C and pulled down with Dynabeads™ Protein G (DB10007, Thermo Scientific) 1 h at 4°C. Finally, the conjugated proteins were eluted using Western blotting.

### Proximity ligation assay (PLA)

The Duolink® (Sigma-Aldrich, MO, United States) PLA was conducted in NRVMs cells, as described before (Hu et al., 2021). The PLA assay is a robust method for detecting proteins located in close proximity (<40 nm) to their native environment. NRVMs were fixed using a blocking solution and incubated with primary antibodies. Following three washes with PBS, secondary antibodies conjugated to PLA probes were applied to the cells and incubated for 1 hour. PLA probes consist of oligonucleotide pairs that, when in close proximity, hybridize to form closed circular DNA, subsequently serving as template primers for rolling-circle amplification. Subsequently, the cell samples on coverslips were mounted with Duolink *in situ* mounting medium with DAPI and examined with confocal microscopy.

### Fluorescence recovery after photobleaching (FRAP)

GJIC was evaluated using the FRAP technique, a widely accepted method (Wahl et al., 2022). NRVMs from both control and TCTP siRNA-knockdown groups were cultured to confluence. Cells were then incubated with 1 μM calcein-AM (Thermo Fisher Scientific, MA, United States), washed with PBS, and imaged using a Zeiss LSM 710 Confocal Microscope (Oberkochen, Germany) equipped with a ×63 objective lens. The Zeiss bleaching application was used to photobleach specific regions of interest, and images were captured every 5 s for 20 min post-bleaching. Fluorescence recovery in the bleached area was analyzed using Zeiss software, and the recovery rate constant was calculated via nonlinear regression analysis using OriginPro version 9.0 (OriginPro Software, MA, United States).

### Scrape loading dye transfer assay

NRVM cultures were subjected to the indicated treatments in a medium containing 130 mM NaCl, 2.8 mM KCl, 1 mM CaCl_2_, 2 mM MgCl_2_, and 10 mM HEPES (pH 7.2) for 5 min at room temperature. Cells were then transferred to a calcium-free medium for 1 min, followed by scraping of the cell monolayer with a scalpel in the presence of 1 mg/mL lucifer yellow (Thermo Fisher Scientific, MA, United States) dissolved in a calcium-free solution. Lucifer yellow was allowed to diffuse into cells for 1 min. The cells were then washed five times with a calcium-containing medium to remove any extracellular dye (Fumagalli et al., 2020). The cells were subsequently fixed in 4% paraformaldehyde, mounted on glass coverslips, and imaged using a Zeiss LSM 710 Confocal Microscope (Oberkochen, Germany) with a ×10 objective lens.

### Western blotting

Tissues from animals or cultured cells were homogenized in lysis buffer (25 mM Tris-HCl [pH 7.5], 150 mM NaCl, 5 mM MgCl_2_, 1% NP-40, 5% glycerol) containing protease inhibitor cocktail (10 mM Na_4_P_2_O_7_, 100 mM NaF, 10 mM Na_3_VO_4_, 20 μg/mL TLCK, 10 μg/mL leupeptin, 1 mM PMSF, 50 U ETI, 2 μg/mL aprotinin) and centrifuged at 15,000 rpm, for 15 min at 4°C. Samples were separated on 10% sodium dodecyl sulfate-polyacrylamide gels and transferred onto nitrocellulose membranes. Primary antibodies were applied overnight at 4°C. Immunoreactive protein bands were visualized using the Clarity Western ECL Substrate kit (Bio-Rad) or Western Lightning ECL Pro kit (Perkin Elmer). Membranes were imaged using an image analyzer (Fujifilm).

### Statistics

All data are expressed as mean ± standard error of the mean (SEM). Data were compared using Student’s t-test for the two groups. Multiple comparisons were made using one-way analysis of variance followed by Tukey’s test or two-way analysis of variance and Bonferroni’s *post hoc* test. For all analytical studies, the criterion of significance was set at P < 0.05.

## Results

### TCTP localized at the intercalated discs

**FIGURE 1:**
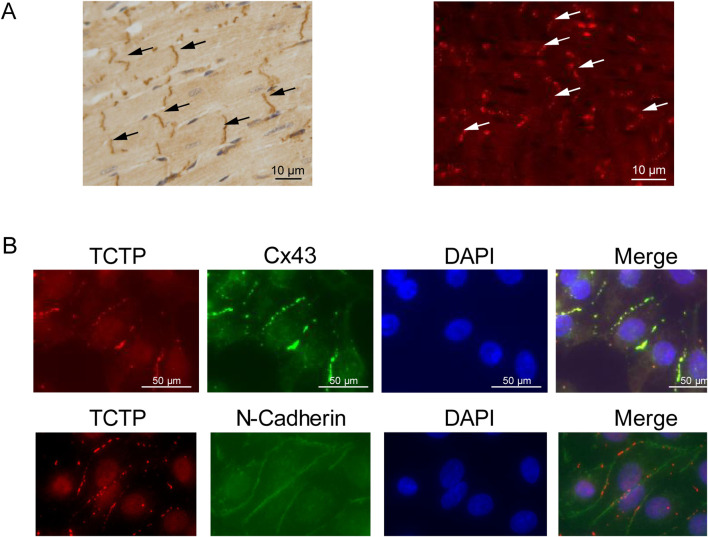
Overlapped distribution of TCTP and Cx43 proteins within cardiac intercalated discs. **(A)** Immunohistochemistry staining (left) and immunofluorescence staining (right) of the anti-TCTP antibody in the left ventricle of mouse hearts show a high concentration of TCTP localized at the intercalated disks (indicated by arrows). Scale bars represent 50 μm. **(B)** Concurrent staining of NRVMs with anti-TCTP (red) and anti-Cx43 (green) antibodies showed colocalization of both signals. A similar outcome was observed when using anti-TCTP (red) and anti-N-cadherin (green) antibodies. Nuclei are stained with DAPI in blue. Scale bars indicate 50 μm. TCTP, Translationally controlled tumor protein; Cx43, Connexin 43; NRVMs, Neonatal rat ventricular myocytes; DAPI, 4′,6-diamidino-2-phenylindole.

We examined the distribution of TCTP in wild-type mouse heart tissue using immunohistochemical staining. TCTP was predominantly localized in the intercalated discs (Figure 1A). To investigate the distribution of TCTP and Cx43 in NRVMs, we immunostained the cells with anti-TCTP and anti-Cx43 antibodies. Both proteins were predominantly expressed at intercellular contacts and co-localized well (Figure 1B).

### TCTP formed complexes and was closely associated with Cx43

**FIGURE 2:**
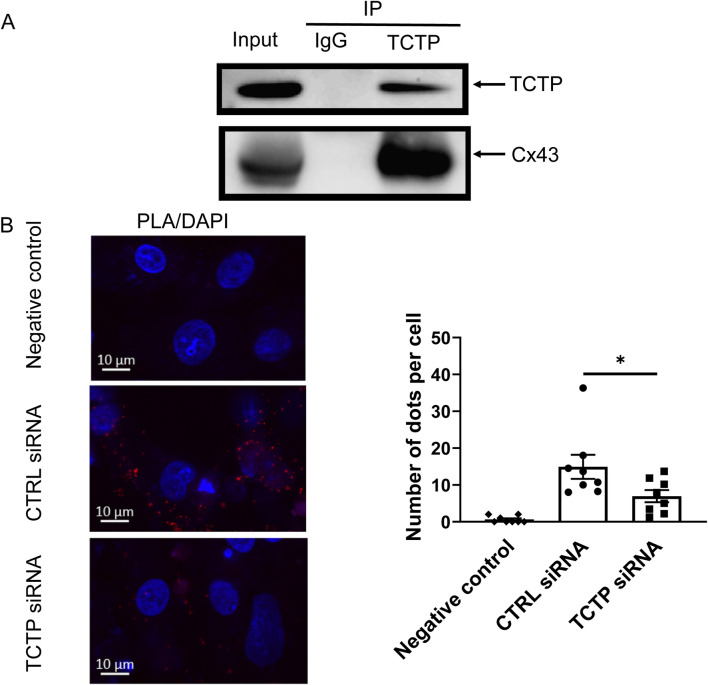
Presence of TCTP/Cx43 protein complex and close localization of TCTP and Cx43 proteins in cardiomyocytes. **(A)** Immunoprecipitation of solubilized lysates from mouse ventricles with anti-TCTP antibody resulted in the co-immunoprecipitation of Cx43, as demonstrated by representative western blots probed with anti-Cx43 antibody. These findings are consistent across three independent experiments. **(B)** Close localization of endogenous TCTP and Cx43 proteins in NRVMs. Representative PLA images of NRVMs transfected with non-targeting siRNA (control siRNA) or with TCTP siRNA. Red dot signals were generated when endogenous TCTP and Cx43 were less than 40 nm. Scale bars indicate 10 μm. Statistical analysis of the PLA signals. Data are displayed as means ± SEM. *P < 0.05 with unpaired t-test (n = 8). TCTP, Translationally controlled tumor protein; Cx43, Connexin 43; NRVMs, Neonatal rat ventricular myocytes; PLA, proximity ligation assay; siRNA, small interfering RNA; DAPI, 4′,6-diamidino-2-phenylindole; SEM, standard error of the mean; CTRL, control; IP, immunoprecipitation; IgG, Immunoglobulin G.

To examine the interaction between TCTP and Cx43 in heart tissue, we performed an immunoprecipitation assay. Cx43 coprecipitated with TCTP from mouse heart tissue extracts (Figure 2A), indicating that TCTP forms complexes with Cx43. Moreover, PLAs revealed that TCTP and Cx43 are closely associated in NRVMs (Figure 2B).

### Colocalization and interaction of TCTP and Cx43 proteins iPSC-CMs

**FIGURE 3:**
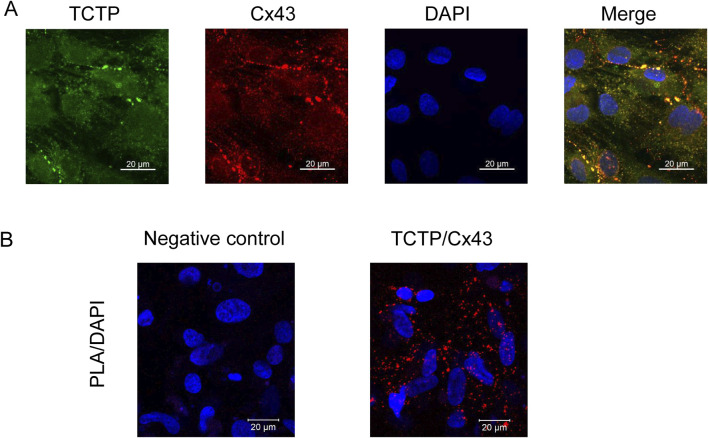
Colocalization and interaction of TCTP and Cx43 proteins in iPSC-CMs. **(A)** Representative confocal images of iPSC-CMs stained with anti-Cx43 (red) and anti-TCTP (green) antibodies. The merged image highlights the colocalization of TCTP and Cx43 at intercellular junctions. Scale bar: 20 μm. **(B)** Proximity localization of endogenous TCTP and Cx43 in iPSC-CMs demonstrated using PLA assay. Representative images show red PLA signals indicating that TCTP and Cx43 are in close proximity (<40 nm). The negative control (left panel) lacks the anti-Cx43 antibody, confirming the specificity of the assay. Nuclei are counterstained with DAPI (blue). Scale bar: 20 μm. TCTP, Translationally controlled tumor protein; Cx43, Connexin 43; iPSC-CMs, Human-induced pluripotent stem cell-derived cardiomyocytes; PLA, proximity ligation assay; DAPI, 4′,6-diamidino-2-phenylindole.

To investigate the relationship between TCTP and Cx43 in human cardiomyocytes, we performed immunostaining and PLA using iPSC-CMs. As shown in Figure 3, co-localization at intercellular contacts and interaction of TCTP and Cx43 proteins were observed in iPSC-CMs.

### TCTP downregulation results in the reduction of Cx43 gap junction channel expression at the intercellular contacts between neonatal rat cardiomyocytes

**FIGURE 4:**
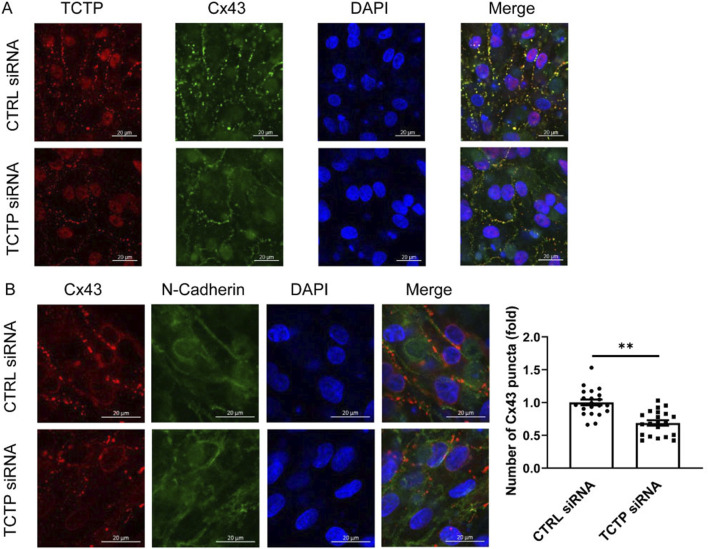
TCTP regulates Cx43 accumulation at intercellular contacts. **(A)** Representative confocal images of NRVMs from control siRNA and TCTP siRNA groups. Double immunolabeled anti-TCTP (red) and anti-Cx43 (green) antibodies showed colocalization. Nuclei are stained blue with DAPI. The lower panel of the cell images indicates reduced colocalization following TCTP siRNA transfection. Scale bars indicate 20 μm. **(B)** Representative confocal images of NRVMs transfected with control siRNA or with TCTP siRNA for 3 days. Cells were stained with anti-Cx43 (red) and anti-N-cadherin (green) antibodies, with the merged image displayed on the right. Note that Cx43 puncta are localized at intercellular junctions, as indicated by N-cadherin staining. Scale bars indicate 20 μm. Quantification of Cx43 accumulation by counting the number of puncta at cell junctions. Two to three randomly selected fields from each culture were used to compare fold changes in Cx43-positive puncta between the control siRNA and TCTP siRNA groups. Data are presented as means ± SEM, expressed as a fold change relative to the control siRNA group (Somekawa et al., 2005). **P < 0.01, analyzed using Student’s t-test (n = 21). TCTP, Translationally controlled tumor protein; Cx43, Connexin 43; siRNA, small interfering RNA; NRVMs, Neonatal rat ventricular myocytes; DAPI, 4′,6-diamidino-2-phenylindole; SEM, standard error of the mean; CTRL, control.

To examine the role of TCTP in the regulation of Cx43 expression, we downregulated TCTP expression using siRNA in NRVMs (Figure 4A). The effect of TCTP siRNA knockdown, which reduced the protein level by more than fivefold, was validated by Western blot analysis (Supplementary Figure S1). Using immunohistochemical staining, we investigated the effect of TCTP downregulation on the number of gap junction plaques expressed at intercellular contacts. Cx43-positive puncta were significantly decreased following TCTP silencing (Figure 4B). These findings indicated that TCTP downregulation decreased Cx43 expression in gap junction plaques between cardiomyocytes.

Notably, no significant difference in Cx43 expression was observed between the control and TCTP-downregulated whole NRVMs lysates (Supplementary Figure S1), suggesting that TCTP regulates the distribution of Cx43 in cells rather than its expression levels.

### TCTP downregulation caused the impairment of GJIC in cardiomyocytes

**FIGURE 5:**
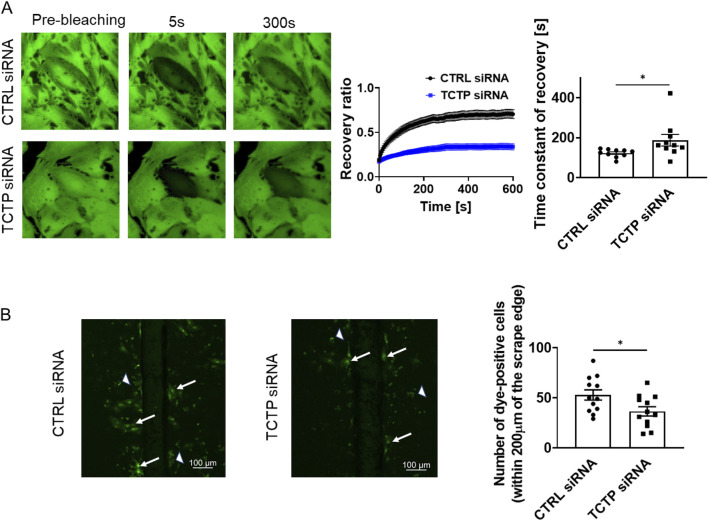
Suppression of gap junctional intercellular communication by TCTP siRNA knockdown in NRVMs. **(A)** Representative fluorescent images showing calcein-AM fluorescence in NRVMs treated with either control siRNA or TCTP siRNA. Cells were loaded with calcein-AM, followed by photobleaching for fluorescence recovery after photobleaching analysis. The fluorescence recovery in bleached cells was tracked for over 600 s post-bleaching and plotted as a function of the recovery time constant (mean ± SEM, n = 10). Quantitative analysis of the recovery time constant using regression fitting. *P < 0.05, determined using Student’s t-test (n = 10) (Rhett et al., 2011; Waxse et al., 2017). **(B)** Representative images from scrape-loading experiments on NRVMs transfected with either control siRNA or TCTP siRNA. Scrape loading demonstrates the extent of dye transfer between neighboring cells via gap junctions. Arrows indicate typical dye-transferred cells, while arrowheads point to cell debris emitting nonspecific fluorescence. Scale bar: 100 μm. Quantitative analysis of the number of dye-positive cells on either side of the scrap and up to 200 μm from the scrap edge (Yang et al., 2019). Data are shown as means ± SEM for control siRNA- or TCTP siRNA-treated NRVMs. *P < 0.05, analyzed using Student’s t-test (n = 12). TCTP, Translationally controlled tumor protein; siRNA, small interfering RNA; NRVMs, Neonatal rat ventricular myocytes; SEM, standard error of the mean; CTRL, control.

Next, to examine the role of TCTP in the regulation of Cx43 function at gap junctions, we evaluated the GJIC between cardiomyocytes using the FRAP assay. The development of GJIC and adherens junctions between adjacent cells has been previously reported in cultured cardiomyocytes (Somekawa et al., 2005; Kizana et al., 2007). The recovery from photobleaching was attenuated by TCTP downregulation with siRNA in NRVMs (Figure 5A). Additionally, scrape-loading dye transfer was decreased by TCTP silencing (Figure 5B), indicating that TCTP expression plays an important role in GJIC maintenance.

### TCTP-overexpression protects against the development of catecholamine-induced ventricular arrhythmias in mice

**FIGURE 6:**
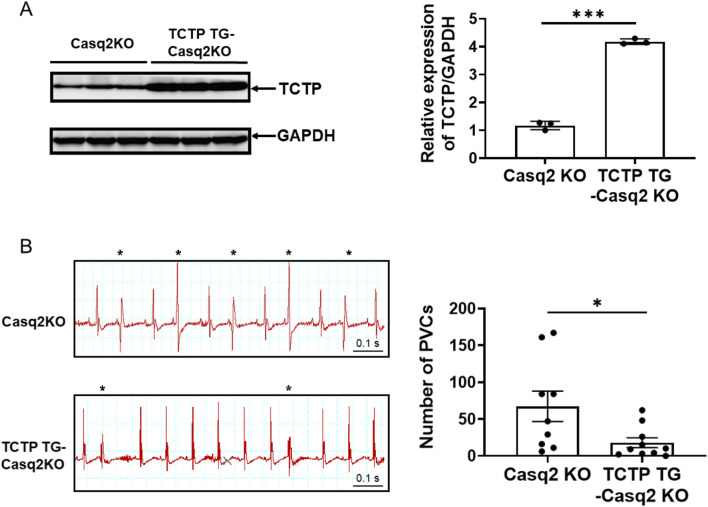
TCTP overexpression reduces catecholamine-induced ventricular arrhythmias. **(A)** Representative Western blot and quantification of total Cx43 protein levels in the left ventricles of Casq2 KO and TCTP TG-Casq2 KO mice, indicating a significant increase in Cx43 expression in TCTP transgenic mice. Protein levels were normalized to GAPDH (n = 3, ***P < 0.001, analyzed with Student’s t-test). **(B)** Representative surface ECG (lead II) recordings from Casq2 KO and TCTP TG-Casq2 KO mice following catecholamine stimulation via intraperitoneal injection of isoproterenol (ISO) (3 mg/kg). The asterisk marks a premature ventricular contraction (PVC). ECGs were recorded for 20 min post-ISO injection, and the total number of PVCs was quantified (n = 9–10, *P < 0.05). Data are presented as means ± SEM. Statistical analysis was performed using Student’s t-test. TCTP, Translationally controlled tumor protein; Cx43, Connexin 43; Casq2 KO, calsequestrin 2 gene knockout; ECG, electrocardiogram; TG, transgenic; GADPH, Glyceraldehyde-3-phosphate dehydrogenase; SEM, standard error of the mean.

To investigate the role of TCTP expression in the development of arrhythmias, we generated cardiomyocyte-specific TCTP-overexpressing mice with a Casq2 KO background (TCTPTG-Casq2 KO), a mouse model of ventricular arrhythmias. In TCTPTG-Casq2 KO mice, TCTP expression levels in ventricular tissue were elevated (Figure 6A). Remarkably, sympathetic activation-induced development of PVCs was attenuated in TCTPTG-Casq2 KO mice compared to Casq2 KO mice (Figure 6B).

## Discussion

In this study, we examined the role of TCTP in gap junctions for the first time.

The present study revealed the following: TCTP is predominantly expressed in the intercalated discs of mouse heart tissue; TCTP is located close to Cx43 in cardiomyocytes; TCTP silencing reduced the formation of Cx43 gap junction channels at the intercellular contacts between cardiomyocytes; TCTP silencing significantly attenuates GJIC in cardiomyocytes; and in cardiomyocytes-specific TCTP overexpressing mouse, the development of catecholamine-induced ventricular arrhythmia was attenuated.

Although TCTP has been demonstrated to be expressed in plasma membranes, there has been no report on the TCTP functions at gap junctions. First, we found that TCTP was predominantly expressed in intercalated discs in the mouse heart tissue, suggesting that TCTP could exert its main functions there.

Next, we showed that TCTP forms complexes with Cx43, a critical component of gap junction channels in the heart. Additionally, the proximity ligation assay revealed that TCTP and Cx43 colocalized at the plasma membrane in the proximity of less than 40 nm. Although whether TCTP directly interacts with Cx43 remains unclear, their close interaction suggests a functional relationship between them.

Interestingly, we found that TCTP silencing resulted in the reduction of Cx43 gap junction channels at the intercellular contacts between cardiomyocytes, indicating that TCTP plays an important role in the regulation of Cx43 gap junction channel formation in cardiomyocytes. The number of gap junction channels at the intercellular contacts is thought to be one of the most important factors affecting GJIC (Luke and Saffitz, 1991; Rodreguez-Sinovas et al., 2021). The maintenance of Cx43 at the intercalated disc may be important in the prevention of cardiac disorders, including arrhythmias (Kieken et al., 2009; Zheng et al., 2023; Zhong et al., 2022). A reduction in Cx43 expression has been reported to cause arrhythmia in mice (Danik et al., 2004; van Rijen et al., 2004; Jansen et al., 2012b). In the human heart, under pathological conditions, including cardiac hypertrophy, ischemia, and dilated cardiomyopathy, reduction of Cx43 at the intercalated discs has been reported (Bruce et al., 2008; Kaprielian et al., 1998; Peters et al., 1993; Salameh et al., 2009). During the development of heart failure, electrical cardiac remodeling, including abnormal expression of Cx43 gap junction channels, is believed to contribute to arrhythmogenesis (Fontes et al., 2012). Importantly, the development of fatal arrhythmias is a significant determinant of the prognosis of patients with heart failure (Zipes and Wellens, 1998). On the other hand, reduced TCTP expression in the hearts of patients with heart failure was reported (Chunhacha et al., 2021). Correctively, it is speculated that the maintenance of TCTP expression could be a strategy for the prevention of arrhythmias in patients with heart failure.

Next, we demonstrated that TCTP is involved in the regulation of GJIC in cardiomyocytes. TCTP silencing significantly attenuated GJIC, accompanied by a reduction in Cx43 gap junction channels at the intercellular contacts. Previous studies have reported several proteins that interact with Cx43 and regulate gap junction functions (Sorgen et al., 2018). Some of the proteins promote GJIC, whereas others inhibit it. In line with the inhibitory effect of TCTP silencing on Cx43 expression at intercellular contacts, TCTP downregulation led to the impairment of GJIC between cardiomyocytes. GJIC has been implicated in crucial pathophysiological processes, including the propagation of action potentials and harmful molecules (e.g., Na^+^) in ischemia/reperfusion injury. Thus, GJIC needs to be appropriately regulated, depending on the situation, to preserve physiological functions and prevent diseases including arrhythmias. Our observations suggested that TCTP plays an important role in maintaining proper GJIC.

To explore the role of TCTP in the development of arrhythmias, we examined the effects of cardiomyocyte-specific TCTP overexpression in Casq2 KO mice, a mouse model of ventricular arrhythmia. Interestingly, TCTP overexpression in cardiomyocytes attenuates their vulnerability to ventricular arrhythmias. It could be speculated that, the GJIC might function more appropriately to maintain normal cardiac conduction in TCTP overexpressing Casq2 KO cardiomyocytes compared with the control. Although the detailed mechanisms remain to be elucidated, these findings indicate that TCTP expression is important in preventing arrhythmogenesis. The agents that affect TCTP expression level including sertraline (Tuynder et al., 2004) and dihydroartemisinin (Cai et al., 2019; Fujita et al., 2008) may influence arrhythmia vulnerability. Further studies using TCTP deficient mice or these agents will be required to evaluate the role of TCTP in the regulation of GJIC and the development of arrhythmias. In contrast to TCTP overexpression, TCTP silencing in cardiomyocytes has been suggested to cause heart failure (Cai et al., 2019; Chunhacha et al., 2021). The maintenance of TCTP expression in cardiomyocytes might be important in the prevention of cardiac diseases.

In addition to arrhythmogenesis, Cx43 also plays an important role in the regulation of cell death. Numerous reports have suggested that Cx43 is involved in the regulation of cell death inside and outside gap junctions (Rodreguez-Sinovas et al., 2021). Furthermore, in the cardiovascular system, mitochondrial Cx43 has been implicated in the development of heart failure (Wei et al., 2022). Contrastingly, TCTP is known as one of the anti-apoptotic proteins. In a previous study, we showed that TCTP protects against doxorubicin (DOX)-induced cardiomyocyte death through a Bcl-2 adenovirus E1B 19-kDa-interacting protein 3 (Bnip3) -dependent mechanism (Cai et al., 2019). Bnip3 is a pro-apoptotic member of the Bcl-2 family of proteins that function in the mitochondria (Dhingra et al., 2014). Interestingly, in our study, the close proximity colocalization of TCTP and Cx43 was observed not only at the plasma membrane but also at intracellular sites (Figures 2B, 3B), suggesting that TCTP might affect Cx43 function in intracellular organelles. It has been speculated that these proteins may work together to regulate cellular viability at mitochondria.

In this study, we performed *in vivo* experiments using male mice. The iPSC-CMs we used were developed from cells of a male donor. Therefore, further studies will be required to assess the gender differences.

TCTP and Cx43 are multifunctional proteins that are thought to be involved in crucial biological processes, including cell growth, cell death, development, vascular tonus, and allergic process (Zhang et al., 2023; Swartzendruber et al., 2020; Uzu et al., 2018). In particular, ongoing investigations are actively exploring the alterations of Cx43’s expression, its intricate localization within cardiac tissues, and the specific post-translational modifications that contribute to its crucial role in maintaining normal cardiac function (Basheer and Shaw, 2016; Leybaert et al., 2023). The findings obtained in this study indicate that these two proteins might influence each other in these physiological and pathological processes. Elucidation of the precise molecular mechanisms underlying their interaction, such as identifying the binding site or key amino acids mediating the TCTP-Cx43 interaction through site-directed mutagenesis, may facilitate the development of novel therapeutic strategies for clinically important diseases.

## Conclusion

These findings indicate that TCTP regulates Cx43 gap junction channel formation at the intercellular contacts. TCTP interacts with Cx43 and plays an important role in GJIC. Thus, TCTP may be a therapeutic target for preventing Cx43-related pathogenesis.

## Data Availability

The original contributions presented in the study are included in the article/Supplementary Material, further inquiries can be directed to the corresponding author.

## Abbreviations

Casq2 KO, calsequestrin 2 gene knockout; CTRL, control; Cx43, Connexin 43; DAPI, 4′,6-diamidino-2-phenylindole; DOX, doxorubicin; ECG, electrocardiogram; EDTA, ethylenediaminetetraacetic acid; FRAP, Fluorescence recovery after photobleaching; GADPH, Glyceraldehyde-3-phosphate dehydrogenase; GJIC, Gap junctional intercellular communication; IgG, Immunoglobulin G; IP, immunoprecipitation; iPSC-CMs, Human-induced pluripotent stem cell-derived cardiomyocytes; ISO, isoproterenol; NRVMs, Neonatal rat ventricular myocytes; PBS, Phosphate-buffered saline; PLA, proximity ligation assay; PVC, Premature ventricular contraction; SD, standard deviation; SEM, standard error of the mean; siRNA, small interfering RNA; TCTP, Translationally controlled tumor protein; TG, transgenic.
