# Supplementary material for: Translationally controlled tumor protein interacts with connexin 43 and facilitates intercellular coupling between cardiomyocytes
Source: Front Cell Dev Biol. 2025 Mar 20;13:1549063. doi: 10.3389/fcell.2025.1549063 (PMC11965915; doi:10.3389/fcell.2025.1549063)
Supplement: Supplementary file 1 [file DataSheet1.pdf]

Supplementary Figures

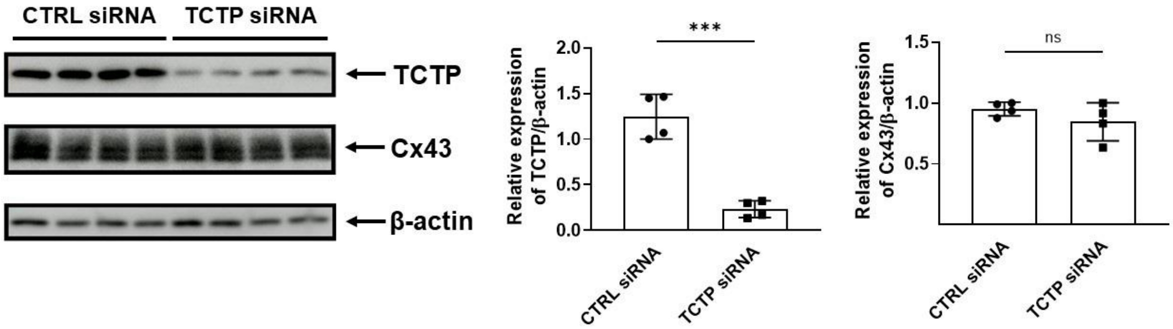

Supplementary Figure 1. TCTP siRNA knockdown does not affect total Cx43 expression in NRVMs. Western blot analysis and quantification of total Cx43 protein levels in NRVMs treated with TCTP siRNA. Results indicate no significant difference in Cx43 expression compared to the control group. Protein levels are normalized to β-actin. Statistical analysis was conducted using an unpaired t-test (n = 4; ***P < 0.001; NS: not significant). CTRL, control; siRNA, small interfering RNA; TCTP, Translationally controlled tumor protein; Cx43, Connexin 43.
